# Post-Translational Changes in Serum Albumin in Patients with Alcohol-Associated Hepatitis

**DOI:** 10.3390/ijms27031503

**Published:** 2026-02-03

**Authors:** Jonathan Montomoli, Maurizio Baldassarre, Thomas Damgaard Sandahl, Marina Naldi, Emilie Glavind, Enrico Pompili, Peter Jepsen, Francesco Palmese, Paolo Caraceni, Hendrik Vilstrup, Marco Domenicali

**Affiliations:** 1 Department of Hepatology and Gastroenterology, Aarhus University Hospital, 8200 Aarhus, Denmark; jonathan.montomoli@gmail.com (J.M.); thomsand@rm.dk (T.D.S.); pj@clin.au.dk (P.J.); vilstrup@clin.au.dk (H.V.); 2 Department of Anesthesia and Intensive Care, Infermi Hospital, Romagna Local Health Authority, 47921 Rimini, Italy; 3 Unit of Semeiotics, Liver and Alcohol-Related Diseases, IRCCS Azienda Ospedaliero-Universitaria di Bologna, 40138 Bologna, Italy; paolo.caraceni@unibo.it; 4 Department of Pharmacy and Biotechnology, Alma Mater Studiorum University of Bologna, 40126 Bologna, Italy; marina.naldi@unibo.it; 5 Center for Applied Biomedical Research (C.R.B.A.), Alma Mater Studiorum University of Bologna, 40126 Bologna, Italy; 6 Department of Medicine, Regional Hospital Horsens, 8700 Horsens, Denmark; emilie.glavind@auh.rm.dk; 7 Department of Medical and Surgical Sciences, Alma Mater Studiorum University of Bologna, 40138 Bologna, Italy; enrico.pompili3@unibo.it (E.P.); m.domenicali@unibo.it (M.D.); 8 Internal Medicine Unit Addressed to Frailty and Aging, Department of Primary Health Care, S. Maria delle Croci Ravenna Hospital, AUSL Romagna, 48121 Ravenna, Italy; francesco.palmese2@unibo.it

**Keywords:** alcohol-associated liver disease, human serum albumin, albumin post-translational modifications, prognosis, mass spectrometry

## Abstract

Post-translational modifications of human serum albumin (HSA) have been described in patients with liver disease. This prospective cohort study aimed to characterize HSA microheterogeneity in hospitalized patients with alcohol-associated hepatitis (AH) and investigate its clinical relevance. We analyzed HSA isoforms by mass spectrometry in 49 patients with AH (at admission and day 14) and 20 healthy controls. Survival at 30, 90, and 365 days was assessed. Differences in HSA isoform abundance were compared between controls and AH patients, as well as between 90-day survivors and non-survivors. AH patients (69% male, median age 53 years) exhibited a significantly different HSA form profile compared to controls, with a lower amount of native HSA and higher oxidized forms. Native HSA negatively correlated with total HSA concentration (R = −0.47, *p* < 0.001). The relative amount of native HSA increased non-significantly from admission to day 14, but its estimated concentration increased significantly (8.8 vs. 12.0 g/L, *p* = 0.005). There were no significant differences in HSA forms between 90-day survivors and non-survivors at admission or day 14. Patients with AH exhibit extensive post-translational modifications of HSA compared to healthy individuals. While HSA forms changed during early hospitalization, they did not significantly correlate with short-term mortality in this cohort.

## 1. Introduction

Human serum albumin (HSA) is the most abundant circulating protein and the main determinant of plasma oncotic pressure, thus being the main modulator of fluid distribution between body compartments. During the past two decades a better understanding of HSA structure has led to the concept that HSA is a multifunctional protein carrying other important biological activities independent from its oncotic power [1]. Among non-oncotic functions, transport of endogenous and exogenous compounds, free radical scavenging, modulation of inflammatory and immunological responses, regulation of capillary permeability and hemostasis are the most important [1].

To date, the definition of hypoalbuminemia is based only on HSA absolute concentration, and most of the existing studies showed that a decrease in HSA is significantly associated with worse prognosis [2,3]. However, besides the reduced plasma concentration, the HSA molecule in patients with advanced cirrhosis as well as in other acute and chronic conditions, presents a series of structural and functional alterations involving several molecular sites [4]. Because of the progressive accumulation of these alterations, the native, fully preserved HSA form [5] is also reduced [6,7]. Such alterations are presumably the results of the pro-oxidant and pro-inflammatory microenvironment that characterizes patients with advanced cirrhosis [8] as well as those with alcohol-associated hepatitis (AH) [9]. Excessive alcohol consumption, independent of the presence of cirrhosis, is associated with an increased generation of reactive oxygen species and systemic oxidative stress, which can damage or cause complete degradation (i.e., peroxidation) of essential complex molecules [10]. Consistently, in AH patients, the amount of oxidized albumin was found to correlate significantly with the extent of systemic oxidative stress and to play an active role in the pathophysiology of the disease [11,12]. Nevertheless, to the best of our knowledge, data regarding the possible prognostic and clinical relevance of HSA post-translational changes in these patients are lacking. Therefore, the present study aimed to describe HSA microheterogeneity in patients hospitalized with AH, based on the hypothesis that these post-translational modifications are driven by the complex interplay between severe oxidative stress, the intense systemic inflammatory response, and the metabolic derangements characteristic of this condition [13]. Furthermore, we also investigated the impact on short-term survival of HSA microheterogeneity in these patients.

## 2. Results

### 2.1. Study Population

The study population consisted of 49 patients with AH. The median age of patients was 53 (IQR 48–58), and 34 (70%) were male. In the control group consisting of 20 healthy subjects, the median age was 43 (IQR 39–47), and 8 (40%) were male. Clinical and laboratory information regarding patients with AH at admission is reported in Table 1. Overall, 11 patients did not have any of the complications recorded at admission, 19 had one complication, 14 had two complications, and 5 had three complications. Ascites was the most prevalent complication at hospital admission, being present in approximately two-thirds of the patients (Table 1).

### 2.2. HSA Microheterogeneity

**Figure 1:**
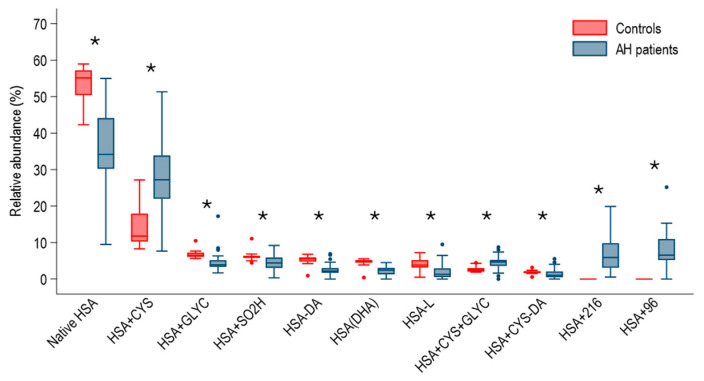
Boxplot comparing the relative amount of human serum albumin (HSA) forms at hospital admission among patients with alcohol-associated hepatitis (AH, n = 49) and healthy subjects (n = 20). Comparisons with a *p*-value < 0.05 are indicated with *. N-terminal truncated HSA (HSA-DA), C-terminal truncated HSA (HSA-L), Cysteinylated and N-terminal truncated HSA (HSA + Cys-DA), HSA in which a cysteine is converted into dehydroalanine (HSA-DHA), Native form of HSA (Native HSA), Sulfinylated HSA (HSA + SO2H). HSA with unidentified modification (HSA + 96), Cysteinylated HSA (HSA + Cys), glycated HSA (HSA + Glyc), HSA with unidentified modification (HSA + 216), Cysteinylated and glycated HSA (HSA + Cys + Glyc).

As expected, patients with AH had a completely different HSA profile than healthy subjects (Figure 1). Namely, the relative amount of native HSA, HSA-DA, HSA-L, HSA + CYS-DA, HSA-DHA, HSA + SO2H, and HSA + GLYC was lower in patients with AH than in healthy subjects. HSA + 96 and HSA + 216 forms were detected, respectively, in 46 and 48 of the 49 patients with AH, while they were absent in the entire group of healthy subjects. Finally, the HSA-CYS and the HSA + CYS + GLYC were significantly higher among patients with AH than among healthy subjects (Figure 1).

When looking at the relative amount of albumin forms in comparison to the concentration of HSA at hospital admission, a negative correlation was present both for native HSA (R = −0.47; *p*-value < 0.001) and for HNA2 (R = −0.41; *p*-value: 0.003). A trend toward positive correlation with HSA concentration was found for HNA1 (R = 0.27, *p*-value = 0.058).

When looking at the changes in HSA between day 0 and 14, a non-statistically significant increase in the relative amount of native HSA was present (Table 1, *p*-value 0.3). Such an increase turned statistically significant when the comparison was performed considering the estimated concentration of native HSA (day 0: 8.8 (SD: 2.5) vs. day 14: 12.0 (2.5) g/L, *p*-value: 0.005). In contrast, there was no difference between day 0 and 14 when considering the HMA, HNA1, and HNA2 forms. Finally, no significant differences in the amount of HMA, HNA1, and HNA2 forms were found among patients treated with anti-inflammatory drugs in the first 14 days from admission compared to those not treated (*p* > 0.05).

### 2.3. Outcomes

**Figure 2:**
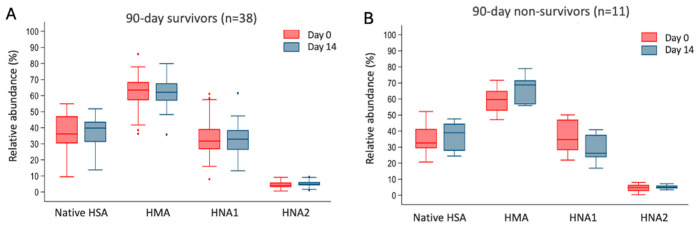
Boxplots comparing the relative amount of native human serum albumin (HSA), human mercaptalbumin (HMA), reversibly oxidized human non-mercaptalbumin-1 (HNA1), and irreversibly oxidized human non-mercaptalbumin-2 (HNA2) at the hospital admission (day 0) and at 14 days (day 14) among patients with alcohol-associated hepatitis in 90-day survivors (**Panel A**) and non-survivors (**Panel B**). None of the comparisons reach a statistically significant *p*-value < 0.05.

Thirty-day, 90-day, and 1-year survival among patients with AH was 90% (81–99), 78% (67–90), and 73% (62–87). At hospital admission, the MELD and the GAHSs significantly differed among 90-day survivors and non-survivors (Table 1). Accordingly, INR and bilirubin were higher among 90-day non-survivors than survivors, while HSA concentration at admission was similar (Table 1). Notably, C-reactive protein was lower among non-survivors than survivors likely as a result of the more compromised synthetic activity of the liver [14] (Table 1). There was no difference among the relative amount of native HSA and albumin forms subgroups between 90-day survivors and non-survivors neither at hospital admission nor on day 14 (Table 1). The changes in the relative amount of native HSA, HMA, HNA1, and HNA2 forms among 90-day survivors and non-survivors and among day 0 and 14 are shown in Figure 2. Although there was a trend in the changes in native HSA and HNA1 among non-survivors (*p*-value 0.06), none reached statistical significance.

**Figure 3:**
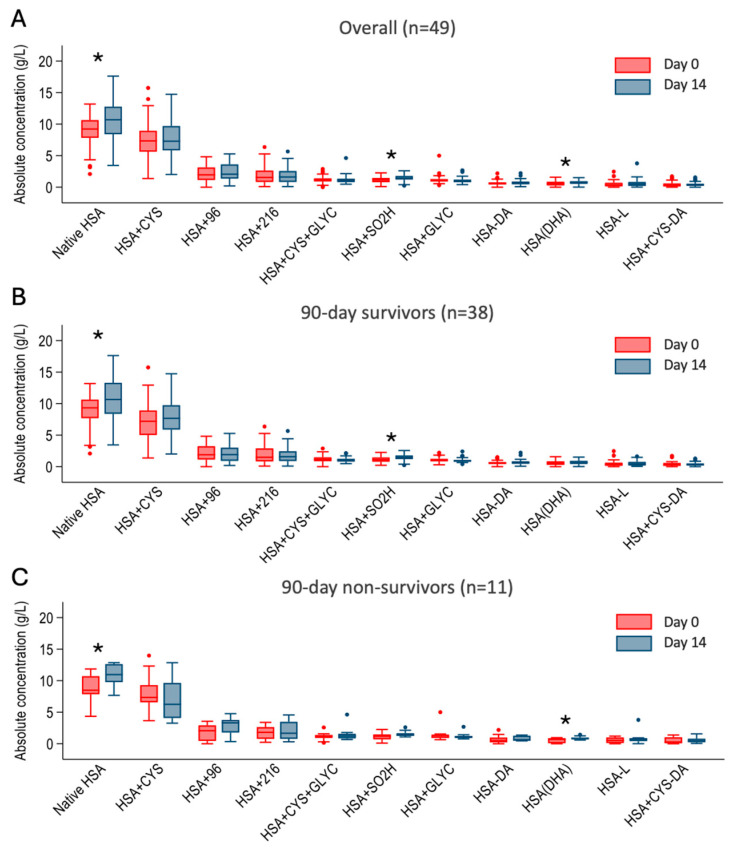
Boxplot comparing calculated concentration of albumin forms (in g/L) at day 0 and day 14 after admission among patients with alcohol-associated hepatitis overall (**Panel A**) and among 90-day survivors (**Panel B**) and non-survivors (**Panel C**). Comparisons with a *p*-value < 0.05 are indicated with *. N-terminal truncated HSA (HSA-DA), C-terminal truncated HSA (HSA-L), Cysteinylated and N-terminal truncated HSA (HSA + Cys-DA), HSA in which a cysteine is converted into dehydroalanine (HSA-DHA), Native form of HSA (Native HSA), Sulfinylated HSA (HSA + SO2H). HSA with unidentified modification (HSA + 96), Cysteinylated HSA (HSA + Cys), glycated HSA (HSA + Glyc), HSA with unidentified modification (HSA + 216), Cysteinylated and glycated HSA (HSA + Cys + Glyc).

When looking at the trend of the estimated concentration of the individual albumin forms overall and among 90-day survivors and non-survivors, native HSA increased significantly from day 0 to day 14 in both groups (Figure 3). Among the other forms, there were no major differences in the changes in the estimated concentration from day 0 to 14 except for the HSA + SO2H in the survivors and HSA-DHA in non-survivors (Figure 3). None of the estimated concentrations of the forms reached a difference that was statistically significant when compared between 90-day survivors and non-survivors.

## 3. Discussion

The present study provides a comprehensive analysis of HSA microheterogeneity in patients with AH, offering novel insights into the complex interplay between liver dysfunction, oxidative stress, and albumin modifications. Our findings reveal a markedly different HSA form profile in AH patients compared to healthy subjects, characterized by lower levels of native HSA and several other forms, coupled with the emergence of unique forms (HSA + 96 and HSA + 216) not detected in healthy individuals. Intriguingly, while we observed these distinct alterations in HSA microheterogeneity, we did not find significant differences in the relative amount of HSA forms between 90-day survivors and non-survivors, either at admission or after 14 days of hospitalization. This suggests that the prognostic value of HSA modifications in AH may be more nuanced than initially hypothesized.

The findings of our study on post-translational modifications of serum albumin in patients with AH align with and expand upon previous research in this area. Das et al. demonstrated that hyperoxidized albumin in patients with severe alcohol-associated hepatitis (SAH) modulates neutrophils to induce oxidative stress and inflammation [11]. Our results corroborate their findings of increased oxidized albumin forms, particularly human non-mercaptalbumin 2 (HNA2), in patients with AH.

Similarly, our observations are consistent with those of Bhat et al., who showed that hyperoxidized albumin plays a significant role in platelet activation and alteration of platelet phenotype in SAH [12]. Their study, like ours, noted increased levels of oxidized albumin forms, specifically human non-mercaptalbumin 1 (HNA1) and HNA2, in patients with alcohol-related liver disease.

While our investigation focused primarily on characterizing albumin forms showing that albumin dysfunction in AH appears primarily driven by the extensive modification of the Cys34 residue rather than other specific variants, previous studies delved deeper into the functional implications of these modifications. Das et al. demonstrated the role of hyperoxidized albumin in neutrophil activation [11], while Bhat et al. showed that oxidized albumin, particularly HNA2, promotes platelet activation, inflammation, and oxidative stress through the CD36 receptor pathway [12]. These mechanistic insights complement our findings by suggesting potential pathways through which the altered albumin profile we observed might contribute to disease pathophysiology. Interestingly, our study provides novel insights into the temporal changes in albumin forms during hospitalization.

The hypoalbuminemia observed in our cohort is a hallmark of the clinical severity of AH. It is important to note that in these patients, low albumin levels are not merely a result of impaired hepatic synthesis. The systemic pro-inflammatory state characteristic of AH increases capillary permeability, leading to the redistribution of albumin into the extravascular space [11,12]. Furthermore, the intense oxidative stress promotes extensive post-translational modifications, which can accelerate the plasma clearance of the albumin molecule [4].

Moreover, the finding of a negative correlation between total HSA and the relative abundance of native HSA provides additional insight into the relationship between the quantity and quality of the circulating albumin pool. Indeed, in patients where albumin concentration is preserved, implying longer circulatory half-life, the protein remains exposed to systemic oxidative stress for extended periods, leading to the accumulation of oxidized forms. Conversely, in severe hypoalbuminemia, accelerated turnover or loss may paradoxically result in a relative preservation of the native form among the remaining molecules.

This study has several notable strengths. The relatively novel application of mass spectrometry to characterize HSA microheterogeneity in AH patients provides new insights into the complex modifications of this crucial protein in the context of severe liver disease. Additionally, the inclusion of a healthy control group enabled us to establish a clear baseline for normal HSA form distribution, against which the alterations in AH patients could be meaningfully compared. Our study also benefits from the comprehensive clinical data collected, including detailed information on complications and established prognostic scores such as MELD and GAHS. This allowed for a nuanced analysis of the relationship between HSA modifications and clinical outcomes. Furthermore, the follow-up period of up to one year provided valuable data on long-term survival, enhancing our understanding of the potential prognostic implications of HSA alterations.

Some limitations should be considered when interpreting our results. First, the diagnosis of AH was based on well-defined clinical and biochemical criteria, but histological confirmation was not routinely performed. Second, the relatively small sample size, particularly for the subgroup analyses evaluating the relationship with survival or with anti-inflammatory drug treatment, may have limited our ability to detect subtle differences in HSA form distribution. Third, the single-center nature of this study may limit the generalizability of our findings to other populations, although it ensured consistency in patient management. Additionally, the selection of patients with paired samples available at day 0 and day 14 inherently introduced a survivorship bias. Potential confounding factors, such as nutritional status, concurrent medications, or variations in the duration of alcohol abuse, could have also influenced HSA modifications. Regarding the control group, although the 10-year age gap compared to AH patients is unlikely to explain the profound structural modifications observed, it must be acknowledged. Finally, the lack of data on HSA modifications beyond 14 days limits our ability to assess how these changes evolve over the long term. Despite these limitations, our study represents an important step forward in understanding the complex alterations of HSA in AH and lays the groundwork for future, larger-scale investigations into the clinical and prognostic significance of these modifications.

## 4. Materials and Methods

### 4.1. Patients

This study was based on two previous studies (NCT03157388 and NCT01245257). From these two prospective studies we included all patients who had blood samples available at day 0 (admission) and day 14. Patients with AH were admitted to the hospital in the Central Denmark Region in Denmark from January 2009 through May 2013. We followed the patients up to one year.

The study protocol conformed to the 1975 Declaration of Helsinki and was approved by the Central Denmark Region Committees on Health Research Ethics.

The AH diagnosis was established by a recent history of alcohol overuse and a combination of clinical and biochemical features compatible with AH. The following inclusion criteria were used: (i) a diagnosis of AH; (ii) a history of excessive alcohol ingestion (5 units or more per day) until at least 3 weeks before admission; (iii) acute jaundice (developed over at most 2 weeks, serum bilirubin ≥ 4.68 mg/dL); (iv) absence of bile duct obstruction or other causes of liver disease; and (v) age 18 years or older. Exclusion criteria were (i) malignancy; (ii) another inflammatory disease; (iii) ongoing gastrointestinal bleeding; (iv) active infection; and (v) treatment with either prednisolone or pentoxifylline during the preceding 8 weeks before entering this study. The recruitment was carried out in 4 hospitals in the Central Denmark Region in Denmark.

Clinical disease severity was assessed according to the Glasgow Alcohol-associated Hepatitis Score (GAHS) [3]. The clinical status of the patients was also assessed using the Model for End-stage Liver Disease (MELD). All patients were given nutritional support and supplements of minerals and vitamins as standard supportive care. A dietician monitored the patients’ nutritional status and their intake, securing moderate hyper-alimentation (a daily energy consumption of 35–40 Kcal/kg body weight and a daily protein intake of 1.2–1.5 g/kg), in all cases where possible by the oral route, if not attainable then via nasogastric tube, or ultimately parenterally. In addition to standard supportive care, patients with GAHS ≥ 9 were treated orally with either prednisolone (oral 40 mg/day) or pentoxifylline (400 mg t.i.d.) (Trental^®^, Sanofi, Paris, France). All patients were screened for bacterial infections using blood and urine cultures, chest radiography and, when appropriate, ascitic fluid culture and determination of the leukocyte count. Laboratory data were recorded at baseline. Information regarding clinical complications present at the hospital admission was recorded including ascites, infection, renal impairment (defined as creatinine > 1.5 mg/dL), and encephalopathy. Moreover, we reported information about prescription of anti-inflammatory treatments (none, pentoxifylline, or prednisolone) during the interval 0 to 14 days. Blood samples for the analysis of the HSA forms were collected at day 0 and day 14 in EDTA-treated tubes and centrifuged at 3000 rpm for 10 min at 4 °C to separate the plasma. A group of 20 healthy subjects was also enrolled as reference population. All plasma samples were stored at −80 °C in dedicated aliquots and had never been thawed prior to the MS analysis.

### 4.2. Assessments of HSA Microheterogeneity

**Figure 4:**
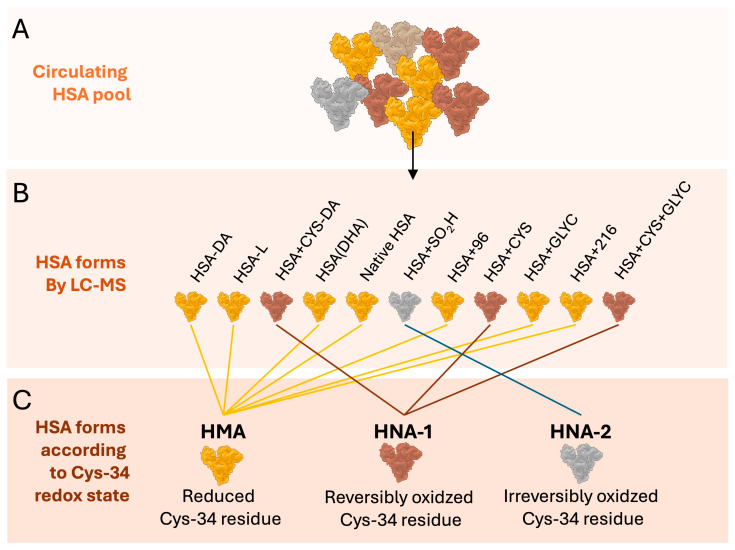
Schematic representation of Human Serum Albumin (HSA) microheterogeneity analysis. HSA isoforms are analyzed in plasma samples using LC-ESI-MS (**Panel A**), which enables the identification and relative quantification of the native HSA along with 10 additional isoforms. Each of these forms is characterized by a specific structural modification or a combination thereof (**Panel B**): N-terminal truncated HSA (HSA-DA), C-terminal truncated HSA (HSA-L), cysteinylated and N-terminal truncated HSA (HSA + Cys-DA), HSA with a cysteine converted into dehydroalanine (HSA-DHA), sulfinylated HSA (HSA + SO2H), HSA with an unidentified modification (HSA + 96), cysteinylated HSA (HSA + Cys), glycated HSA (HSA + Glyc), HSA with an unidentified modification (HSA + 216), and cysteinylated and glycated HSA (HSA + Cys + Glyc). Finally, these isoforms were categorized according to the redox state of the Cys-34 residue (**Panel C**) into: human mercaptalbumin (HMA), non-mercaptalbumin-1 (HNA1), and non-mercaptalbumin-2 (HNA2).

The assessment of HSA structural modification was achieved by applying the analytical approach previously published [15]. The validated analytical method was systematically applied on a Q-ToF Micro quadrupole time-of-flight (Q-TOF) hybrid analyzer (Micromass, Manchester, UK) with a Z-spray electrospray ion source (ESI) for the screening of the protein microheterogeneity in the studied cohort. The HSA forms identified in plasma samples (Figure 4) have been previously described [16]. The abundance of single forms was calculated as the ratio between their fractional intensity and the sum of the intensities of all forms, expressed as percentage. Moreover, an estimate of the serum concentration of each form was computed by multiplying the relative amount of each form by the overall concentration of the HSA obtained from the laboratory. Finally, in order to compare the study findings with some of the existing literature, HSA forms were also classified in three previously described groups according to the redox state of its cysteine 34 residue (Cys-34, Figure 4): (i) human mercaptalbumin (HMA: N-terminal truncated HSA [HSA-DA], C-terminal truncated HSA [HSA-L], HSA in which a cysteine is converted into dehydroalanine [HSA-DHA], Native form of HSA [HSA], HSA with unidentified modification [HSA + 96], glycated HSA [HSA + Glyc], HSA with unidentified modification [HSA + 216]), (ii) reversibly oxidized human non-mercaptalbumin-1 (HNA1: Cysteinylated and N-terminal truncated HSA [HSA + Cys-DA], Cysteinylated HSA [HSA + Cys], Cysteinylated and glycated HSA [HSA + Cys + Glyc]), and (iii) irreversibly oxidized human non-mercaptalbumin-2 (HNA2: Although the HNA2 fraction includes several different HSA forms characterized by the irreversible oxidation of the Cys-34 residue, the sulfinylated HSA [HSA + SO2H] was the only specific form detected by our mass spectrometry method in this cohort) [16,17].

### 4.3. Statistical Analysis

The results were expressed as the median and interquartile range (IQR) unless otherwise specified. Normality was checked graphically using Q–Q plots and by the Shapiro–Wilk test. The Kaplan–Meier technique was used to compute 30-day, 90-day, and 365-day survival and 95% (confidence interval 95% CI) among AH patients. Differences in the relative abundance of each HSA form between control subjects and hospitalized patients with AH and among 90-day survivors vs. non-survivors were evaluated using either a Wilcoxon rank-sum test with continuity correction or Student’s *t*-test according to the data distribution. Variation in the relative abundance of HSA forms between T0 and T14 was evaluated by the Wilcoxon signed-rank test or the paired *t*-test according to data distribution. Given the exploratory and descriptive nature of this study no correction for multiple comparisons was applied.

Comparisons between categorical variables were made by means of the chi-square test or Fisher’s exact test. Homogeneity of variances was assessed by the Levene test, and the Welch’s correction will be applied when appropriate. For all statistical analyses, a fully scripted data management pathway was created within the R environment for statistical computing, version 4.2.2 (R Development Core Team, 2011).

## 5. Conclusions

In conclusion, our study provides novel insights into the complex landscape of albumin modifications in AH, revealing distinct patterns of HSA microheterogeneity that differ markedly from healthy individuals. While our findings did not demonstrate a clear prognostic value for HSA modifications in short-term mortality, they lay crucial groundwork for future investigations. Future studies should validate our findings and aim to better investigate the temporal changes in HSA forms and their correlation with clinical conditions throughout the course of AH. These efforts will help elucidate the potential role of albumin modifications as biomarkers for disease progression and treatment response, potentially leading to improved management strategies for patients with AH.

## Figures and Tables

**Table 1:** Clinical information and human albumin forms relative abundance in patients with alcohol-associated hepatitis (AH) among survivors and non-survivors at 90 days from hospital admission. Abbreviations: Alcohol-associated hepatitis (AH), alanine transaminase (ALT), interquartile range (IQR), human serum albumin (HSA), Human mercaptalbumin (HMA), Human nonmercaptalbumin-1 (HNA1), Human nonmercaptalbumin-2 (HNA2), Model for End-stage Liver Disease (MELD), Glasgow Alcohol-associated Hepatitis Score (GAHS). * Wilcoxon rank-sum test with continuity correction or *t*-test for comparison of continuous variables according to the variable distributions. Chi-squared test for categorical variables among 90-day survivors vs. non-survivors. ^a^ Two missing observations. ^b^ One missing observation. ^c^ Four missing observations.

Characteristics	AH Population N = 49	90-Day Survivors N = 38	90-Day Non-Survivors N = 11	*p*-Value *
Age, median (IQR)	53 (48–58)	52 (47–57)	54 (48–58)	0.611
Male sex, n (%)	34 (69)	27 (71)	7 (64)	0.638
Laboratory at admission, median (IQR):				
- Albumin, g/L	26 (22–30)	26 (22–30)	26 (23–31)	0.622
- C-Reactive protein, mg/dL ^a^	32 (23–45)	34 (25–51)	25 (12–36)	0.016
- Bilirubin, mg/dL	19.1 (11.8–24.5)	17.3 (10.5–22.7)	24.0 (17.3–26.6)	0.080
- ALT, U/L	43 (32–68)	48 (33–70)	37 (28–45)	0.150
- INR	1.9 (1.6–2.2)	1.9 (1.5–2.0)	2.1 (2.0–2.5)	0.028
- Creatinine, mg/dL ^b^	0.71 (0.55–0.99)	0.67 (0.55–0.93)	0.95 (0.70–1.27)	0.111
- Urea, mg/dL ^c^	21 (12–34)	20 (11–33)	22 (13–46)	0.543
- Sodium, mmol/L ^b^	132 (127–135)	133 (127–135)	132 (127–134)	0.873
Clinical complications at admission, n (%)				
- Encephalopathy	16 (33)	9 (24)	7 (64)	0.013
- Ascites	33 (67)	25 (66)	8 (73)	0.666
- Infection	6 (12)	4 (10)	2 (18)	0.495
- Renal impairment ^b^	7 (15)	4 (11)	3 (27)	0.174
Liver scores at admission, median (IQR):				
- MELD ^b^	23 (17–27)	21 (16–26)	27 (23–31)	0.048
- GAHS ^c^	9 (8–10)	9 (8–10)	10 (9–11)	0.026
Anti-inflammatory drug, n (%):				
- Pentoxifylline	34 (69.4)	25 (65.8)	9 (81.8)	0.464
- Prednisolone	4 (8.2)	3 (7.9)	1 (9.1)	1.000
- None	11 (22.4)	10 (26.3)	1 (9.1)	0.415
% of HSA forms at admission, median (IQR)				
- Native HSA	34.2 (30.2–44.1)	36.2 (30.4–46.4)	32.6 (29.7–37.4)	0.523
- HMA	62.3 (56.1–67.9)	63.5 (57.3–68.3)	59.6 (53.3–64.2)	0.337
- HNA1	32.6 (27.2–39.5)	31.7 (26.8–39.1)	34.79 (30.9–43.3)	0.337
- HNA2	4.4 (3.1–5.9)	4.1 (3.2–5.9)	4.8 (2.8–6.2)	0.915
% of HSA forms at day 14, median (IQR)				
- Native HSA	39.3 (31.2–43.7)	39.8 (32.0–43.7)	38.9 (29.7–44.0)	0.775
- HMA	62.2 (56.9–68.7)	62.1 (57.0–67.6)	68.7 (58.5–71.0)	0.145
- HNA1	32.4 (25.2–37.6)	32.9 (26.4–38.2)	26.2 (23.8–35.0)	0.171
- HNA2	4.9 (4.3–6.1)	4.8 (4.2–6.1)	5.0 (4.4–5.9)	0.881

## Data Availability

The data shown in this article are available from the corresponding author upon a reasonable request.

## Abbreviations

The following abbreviations are used in this manuscript:

AH	Alcohol-associated hepatitis
ALT	Alanine transaminase
GAHS	Glasgow Alcohol-associated Hepatitis Score
HMA	Human mercaptalbumin
HNA1	Human nonmercaptalbumin-1
HNA2	Human nonmercaptalbumin-2
HSA	Human serum albumin
HSA + 216	HSA with unidentified modification
HSA + 96	HSA with unidentified modification
HSA + CYS	Cysteinylated HSA
HSA + CYS + GLYC	Cysteinylated and glycated HSA
HSA + CYS-DA	Cysteinylated and N-terminal truncated HSA
HSA + GLYC	Glycated HSA
HSA + SO_2_H	Sulfinylated HSA
HSA-DA	N-terminal truncated HSA
HSA-DHA	HSA in which a cysteine is converted into dehydroalanine
HSA-L	C-terminal truncated HSA
IQR	Interquartile range
MELD	Model for End-stage Liver Disease
SAH	Severe alcohol-associated hepatitis
